# LSN2424100: a novel, potent orexin-2 receptor antagonist with selectivity over orexin-1 receptors and activity in an animal model predictive of antidepressant-like efficacy

**DOI:** 10.3389/fnins.2014.00005

**Published:** 2014-01-28

**Authors:** Thomas E. Fitch, Mark J. Benvenga, Cynthia D. Jesudason, Charity Zink, Amy B. Vandergriff, Michelle M. Menezes, Douglas A. Schober, Linda M. Rorick-Kehn

**Affiliations:** Lilly Research Laboratories, Department of Neuroscience, Eli Lilly and CompanyIndianapolis, IN, USA

**Keywords:** hypocretin, orexin, neuropeptide, OX2 antagonist, antidepressant, DRL, rat, mouse

## Abstract

We describe a novel, potent and selective orexin-2 (OX2)/hypocretin-2 receptor antagonist with *in vivo* activity in an animal model predictive of antidepressant-like efficacy. *N*-biphenyl-2-yl-4-fluoro-*N*-(1H-imidazol-2-ylmethyl) benzenesulfonamide HCl (LSN2424100) binds with high affinity to recombinant human OX2 receptors (*Ki* = 4.5 nM), and selectivity over OX1 receptors (*Ki* = 393 nM). LSN2424100 inhibited OXA-stimulated intracellular calcium release in HEK293 cells expressing human and rat OX2 receptors (*Kb* = 0.44 and 0.83 nM, respectively) preferentially over cells expressing human and rat OX1 (*Kb* = 90 and 175 nM, respectively). LSN2424100 exhibits good exposure in Sprague–Dawley rats after IP, but not PO, administration of a 30 mg/kg dose (AUC_0–6 *h*_ = 1300 and 269 ng^*^h/mL, respectively). After IP administration in rats and mice, LSN2424100 produces dose-dependent antidepressant-like activity in the delayed-reinforcement of low-rate (DRL) assay, a model predictive of antidepressant-like efficacy. Efficacy in the DRL model was lost in mice lacking OX2, but not OX1 receptors, confirming OX2-specific activity. Importantly, antidepressant-like efficacy of the tricyclic antidepressant, imipramine, was maintained in both OX1 and OX2 receptor knock-out mice. In conclusion, the novel OX2 receptor antagonist, LSN2424100, is a valuable tool compound that can be used to explore the role of OX2 receptor-mediated signaling in mood disorders.

## Introduction

The orexin (or hypocretin) family of neuropeptides, termed orexin-A (OXA) and orexin-B (OXB), and the receptors to which they bind, were first identified in the late 1990's (De Lecea et al., 1998; Sakurai et al., 1998). The orexin peptides bind to two G-protein coupled receptors, orexin-1 (OX1) and orexin-2 (OX2), with OXA showing roughly equal affinity for OX1 and OX2 receptors and OXB binding preferentially to OX2 receptors (Spinazzi et al., 2006). Although orexin-containing neurons are predominantly localized in the lateral and posterior hypothalamus, they send widespread projections throughout the neuro-axis (Spinazzi et al., 2006). Orexin has been demonstrated to play a role in mediating feeding (Sakurai et al., 1998), regulation of sleep/wake states (Chemelli et al., 1999), addiction and reward-seeking behavior (Richards et al., 2008; Aston-Jones et al., 2009), monoaminergic transmission (Borgland et al., 2008; Ortega et al., 2012), and stress modulation (Boutrel et al., 2005). That many of these functions are often dysregulated in depression has led researchers to propose that orexins may be involved in the pathophysiology of depression (reviewed in Nollet and Leman, 2013).

The exact role of orexins in modulating mood and depressive disorders has proven more difficult to delineate. Early clinical studies evaluating the potential role of orexin in depressive disorders indicated that depressed patients had a blunted diurnal variation in CSF orexin-A levels accompanied by higher orexin-A levels at night, and that these differences were improved with antidepressant treatment, suggesting a link between disrupted orexin signaling and HPA axis disruption associated with depression (Salomon et al., 2003). However, later studies reported contradictory findings, specifically that CSF orexin-A levels were decreased in depressed patients who had attempted suicide (Brundin et al., 2007). To better understand the role of orexin signaling in depression, some researchers turned to putative genetic animal models of depression, although these studies also produced seemingly contradictory findings. For example, it has been demonstrated that Wistar–Kyoto rats, long-regarded as a genetic model of depression based on enhanced sensitivity to stress and reduced sensitivity to selective serotonin reuptake inhibitors (SSRIs; Lopez-Rubalcava and Lucki, 2000), exhibit reduced numbers of orexin neurons, as well as smaller soma size, lower prepro-orexin mRNA expression, and lower OXA levels in various brain areas (Taheri et al., 2001; Allard et al., 2004). The Flinders Sensitive Line (FSL) is another line of selectively bred animals with a prodepressive phenotype characterized by reduced body weight, reduced sexual behavior, disrupted sleep/wake patterns (particularly REM sleep) and exaggerated immobility responses in the Porsolt Forced swim test (Overstreet and Wegener, 2013). In contrast to results in Wistar–Kyoto rats, the FSL rats were found to have a higher number of OX-immunopositive hypothalamic neurons associated with higher immobility in the forced swim test (Mikrouli et al., 2011). Recently, Nollet et al. (2011) reported that unpredictable chronic mild stress was associated with increased aggression and activation of orexin neurons in the dorsomedial hypothalamus, along with concomitant reductions in the number of OX2, but not OX1, receptors in ventral hippocampus, thalamus and hypothalamus. Interestingly, these effects were reversed by chronic treatment with the SSRI antidepressant, fluoxetine (Nollet et al., 2011). The dual orexin receptor antagonist, almorexant, has also been shown to produce antidepressant-like effects in the chronic unpredictable mild stress model (Nollet et al., 2012). While accumulating evidence suggests that orexin neurotransmission may play a role in modulating mood states, little is known about the molecular mechanisms involved.

A more thorough understanding of the involvement of the orexin system in mediating depression, a complex disorder with multiple behavioral sequelae, would benefit from the availability of precise pharmacological probes from multiple chemical scaffolds. These, in turn, could lead to the development of novel pharmacotherapies targeting the orexin system that could prove beneficial in treating depressive disorders. The OX1 antagonist SB334867 (Smart et al., 2001), and the dual orexin antagonist, almorexant (Brisbare-Roch et al., 2007), have been broadly utilized in preclinical experiments. Although OX2-selective antagonists have been reported in the literature, including TCS-OX2-29 (Hirose et al., 2003), JNJ-10397049 (McAtee et al., 2004), and EMPA (Malherbe et al., 2009), very little has been published on these molecules, especially with regard to antidepressant-like efficacy.

**Figure 1:**
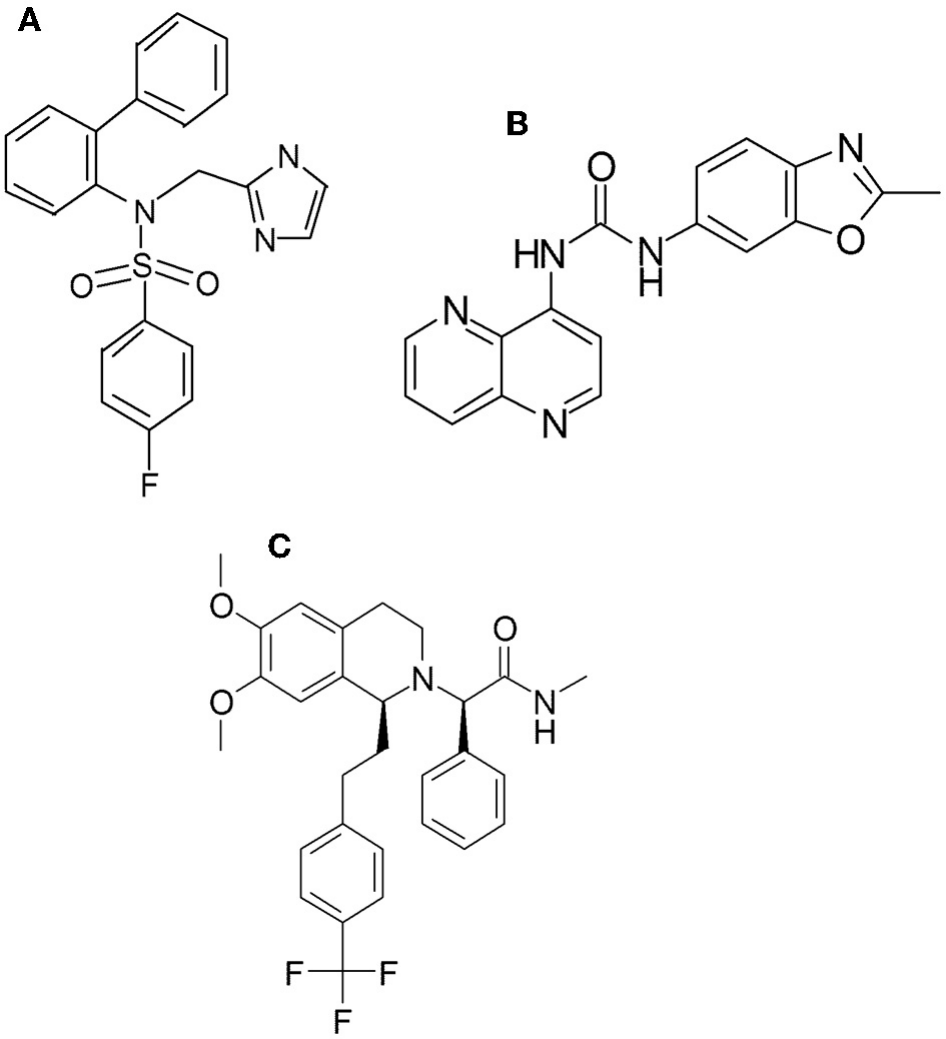
**Chemical structure of LSN2424100 (A), SB334867 (B), and almorexant (C)**.

Here, we describe a novel OX2 receptor antagonist *N*-((1*H*-imidazol-2-yl)methyl)-*N*-([1,1′-biphenyl]-2-yl)-4-fluorobenzenesulfonamide hydrochloride (LSN2424100, Figure 1), and characterize it in terms of its *in vitro* binding affinity, functional selectivity, and pharmacokinetic properties, and further examine its effects on c-fos expression in the rat prefrontal cortex, a brain region implicated in the pathophysiology of depression (Drevets et al., 2008), in response to restraint stress. We then contrast the effects of this compound in an established animal model predictive of antidepressant-like efficacy, the differential reinforcement of low-rate (DRL) schedule of reinforcement, in both rat (DRL-72; O'Donnell et al., 2005), and mouse (DRL-36; Zhang et al., 2009), as well as mice lacking OX1 and OX2 receptors. The DRL model has been pharmacologically validated for detecting antidepressant-like efficacy using clinical antidepressants across multiple pharmacological classes, including tricyclic antidepressants, SSRIs, norepinephrine reuptake inhibitors, and monoamine oxidase inhibitors as indicated by reduced impulsivity, improved response inhibition, and improved response timing (O'Donnell et al., 2005; Zhang et al., 2009).

## Materials and methods

### Drugs and reagents

*N*-((1*H*-imidazol-2-yl)methyl)-*N*-([1,1′-biphenyl]-2-yl)-4-fluorobenzenesulfonamide hydrochloride (LSN2424100), SB334867, and both (S)-almorexant (ACT-078573; Weller et al., 2005) and its inactive enantiomer (R)-almorexant were synthesized at Lilly Research Laboratories (Indianapolis, IN; see Figure 1). LSN2424100 and SB334867 were suspended in a solution of 1% carboxymethylcellulose, 0.25% polysorbate 80, and 0.05% Dow antifoam and injected IP in a volume of 10 ml/kg body weight for mice and 1 ml/kg in rat (LSN2424100 was injected in a 2 ml/kg volume for c-fos studies). (S)-almorexant and (R)-almorexant were suspended in a solution of 1% carboxymethylcellulose, 0.25% polysorbate 80, and 0.05% Dow antifoam and dosed PO in a volume of 2 ml/kg in rat (dosed IP at 10 ml/kg for mice). Imipramine and alprazolam were purchased from Sigma-Aldrich (St. Louis, MO). Imipramine was dissolved in sterile water. Alprazolam was dissolved in 10% 2-hydroxypropyl-β-cyclodextrin. Drugs were mixed fresh on the day of the experiment and doses were calculated based on the free base weight.

### Subjects

Adult Male Sprague–Dawley rats weighing 200–225 g (Harlan, Indianapolis, IN for c-fos experiments) were housed 4 per cage with *ad libitum* access to food (Teklad 4% Rat Diet; Harlan Teklad, Madison, WI) and water (except during experimental sessions) and maintained on a 12 h light:dark cycle (lights on at 0600 h). Rats were acclimated to housing conditions for 4 days, followed by sham dosing once daily for 3 days, prior to the experiment. For DRL experiments, male Sprague–Dawley rats weighing between 300 and 350 g at the beginning of the behavioral experiments (Holtzman, Madison, WI) were housed in pairs. For mouse DRL studies, male C57Bl/6 mice (Taconic Farms, Hudson, NY) or mice lacking either OX1 or OX2 receptors, around 8 weeks of age, were obtained from private breeding colonies at Taconic Farms (Hudson, NY). OX1 and OX2 receptor knockout mice were generated using *in vitro* fertilization of embryos in C57Bl/6 female mice with sperm harvested from male mice obtained from the University of Texas Southwestern Medical Center (Dallas, TX), and backcrossed for at least 10 generations. Mice and rats were housed in separate colony rooms, which were maintained at 22°C and 60% relative humidity. For rat DRL experiments, water was available for a 20-min period following the daily behavioral session. Based on recommendations from the animal care and use committee, the mouse DRL assay was developed using food deprivation rather than water deprivation. Mice had free access to water except during experimental sessions, were maintained at 85% of free-feeding weight, and received 1 h of free-feeding after each experimental session. All experiments were conducted during the light cycle and in compliance with the Guide for the Care and Use of Laboratory Animals under protocols approved by a local animal care and use committee.

### Radioligand binding

Recombinant human OX1 or OX2 receptors were stably expressed in HEK293 cells and grown in DMEM/F-12 (3:1) supplemented with 5% FBS, 20 mM HEPES, 100 ug/ml Penn/Strep, and 500 ug/ml geneticin. Briefly, the membranes were isolated by homogenizing cell pellet in 30 ml (w/v) 50 mM Tris buffer (pH 7.4) containing Roche Complete EDTA free protease tablets. Membranes were incubated with ~0.25 nM [^125^I]-Orexin A (PerkinElmer, Inc., Waltham, MA) for 90 min at 22°C in polystyrene 96-deep well plates. All binding studies were conducted at a final volume of 200 μl. The assay buffer contained 25 mM HEPES, 2.5 mM CaCl_2_, 1.0 mM MgCl_2_, 0.5% BSA, and 0.125% BSA (pH 7.4). To generate binding affinity (*Ki*), 11 different compound concentrations were incubated with ~15 μg of OX1 membrane or ~50 μg of OX2 membrane in the presence of [^125^I]-Orexin A. Compounds were solubilized to make a 10 mM stock in DMSO then diluted to 40 μM by placing 4 μl into 996 μl binding assay buffer. Then 300 μl of this solution was placed in column B on a Nunc polypropylene 96-well plate (Thermo Fisher Scientific, Inc., Rochester, NY). Serial dilutions were then made using a Biomek 2000 (Beckman Coulter, Inc., Fullerton, CA) from a starting concentration of 40 μM. Non-specific binding was determined in these experiments using 10 μM SB-334867 and LSN2158312 for OX1 and OX2, respectively. All binding was terminated by rapid filtration using a TOMTEC 96-well cell harvester (Hamden, CT) through GF/A filters that had been presoaked with 0.3% polyethyleneimine. The filters were washed with 5.0 ml ice-cold 50 mM Tris buffer (pH 7.4) and air-dried overnight. The dried filters were treated with MeltiLex A (PerkinElmer, Inc., Waltham, MA) melt-on scintillator sheets, and the radioactivity retained on the filters counted using a Wallac 1205 Betaplate (PerkinElmer, Inc., Waltham, MA) scintillation counter. Protein concentrations were measured using Coomassie Protein Plus Assay Reagent (Pierce, Rockford, IL) and serum albumin standards. *Ki*-values from displacement of [^125^I]-orexin-A binding were calculated based on 11-point dilution curves using ActivityBase templates (ID Business Solutions, Ltd., Guildford, Surrey, UK). Reported values are shown as a mean ± the standard error of the mean (s.e.m.).

### Intracellular calcium mobilization

Recombinant human OX1 and OX2 receptors were stably expressed in HEK293 cells, or rat recombinant OX1 and OX2 receptors stably expressed in AV12 cells, and assessed for intracellular calcium mobilization using Fluo-3 dye (Molecular Probes, Eugene, OR). Fluo-3 dye was made at 2.2 mM in equal parts of Pluronic f-127 (Molecular Probes, Eugene, OR) and 100% DMSO. It was further diluted to 8 mM in 2.5 mM probenecid loading buffer which was made as described below. The human cell line was grown in DMEM/F-12 (3:1) supplemented with 10% FBS (heat-inactivated), 20 mM HEPES, 1% Penn/Strep, and 4 μg/ml Blasticidin (35,000 cells/well). The rat cell line was grown in DMEM supplemented with 10% FBS (heat-inactivated), 20 mM HEPES, 1% Penn/Strep, 4 μg/ml Blasticidin, and 1 mM sodium pyruvate (60,000 cells/well). Cells were plated in Biocoat black poly-d-lysine coated clear bottom 96-well plates (Becton Dickinson, Bedford, MA), allowed to attach for 30 min at room temperature, and then grown overnight at 37°C and 5% CO_2_ in a humidified incubator. Probenecid loading buffer was made at 250 mM in equal parts of 1 N NaOH and HBSS. It was further diluted to 2.5 mM in HBSS containing calcium and magnesium +20 mM HEPES. The agonist used in this study was Orexin-A (Bachem/Peninsula Laboratories, San Carlos, CA) dissolved in de-ionized water at a stock concentration of 80 μM. An EC_70_ concentration of orexin A in 0.1% BSA (Sigma Aldrich, St. Louis, MO) in HBSS with calcium and magnesium, was used to challenge the antagonists. Compounds were serial diluted 1:3 from a 10 mM stock in DMSO on a Biomek (Beckman Coulter, Inc., Fullerton, CA) and further diluted in HBSS containing calcium and magnesium +0.04% Bacitracin (USB Corp., Cleveland, OH). The assay was performed with the following steps: (1) growth medium was removed and cells were washed with 30 μl HBSS (with calcium and magnesium) and then removed. Next, 30 μl of dye was added to the wells, and cell plates were incubated in the dark at room temperature for 60 min; dye was removed, cells were washed with 30 μl probenecid loading buffer and removed, and then 50 μl of probenecid loading buffer was added to the wells. Finally, plates were read on a fluorescence imaging plate reader (FLIPR; Molecular Devices, LLC, Sunnyvale, CA) instrument with a 50 μl addition of antagonist; cells were placed in the dark at room temperature for an additional 15 min; then read on FLIPR instrument with a 100 μl addition of an EC_70_ concentration of orexin A (for a total volume of 200 μl). Final concentrations of the test compounds were 20 μM in 1.25% DMSO and 10 μM in 0.625% DMSO for the two different FLIPR reads, respectively. *Kb*-values were calculated based on 10-point dilution curves using ActivityBase templates (ID Business Solutions, Ltd., Guildford, Surrey, UK). Reported values are shown as a mean ± the standard error of the mean (s.e.m.).

### Rat exposure and unbound fraction

Two male cannulated rats were administered a single 30 mg/kg intraperitoneal (IP) or 30 mg/kg oral (PO) dose of LSN2424100 to determine the pharmacokinetic parameters. Plasma samples were collected at 0.5, 1, 1.5, 2, 4, and 6 h post-dose and analyzed by liquid chromatography coupled to tandem mass spectral detection (LC-MS/MS) to determine the concentrations of LSN2424100. Two cohorts of rats were used in each study, with samples taken from one cohort at 0.5, 1, and 2 h post-dose, and samples taken from the second cohort at 1.5, 4, and 6 h post-dose. The first cohort of animals was sacrificed at 2 h post-dose, and the second at 6 h post-dose to allow for collection of brain samples. The plasma and brain binding of LSN2424100 were determined by equilibrium dialysis at 1 μM which were used to calculate unbound concentrations in brain and plasma at 2 h post-dose (Zamek-Gliszczynski et al., 2011).

### Restraint-stress-induced c-Fos activation

Beginning at 0830 h on the day of the study, groups of rats (*n* = 8 per group) received IP injections of vehicle, 30 mg/kg LSN2424100, or 3 mg/kg alprazolam in a counter-balanced manner and were returned to the home cage. Thirty min later, rats were restrained in a rigid plastic flat-bottom restraint tube (Braintree Scientific, Inc.; Braintree, MA, USA) for 20 min. After the 20 min stress period, rats were moved to a new cage and housed individually. Rats were sacrificed 2 h after stress onset. Separate groups of control animals (*n* = 6 – 8 each) received either vehicle or 30 mg/kg LSN2424100 and were returned to their home cage until sacrifice. Immediately after sacrifice, brains were removed, the medial prefrontal cortex (mPFC), a brain area implicated in the pathophysiology of depression, was dissected from both hemispheres and frozen in 1.5 ml centrifuge tubes in dry ice, and stored at −80°C for later analysis.

### c-Fos elisa analysis

Frozen mPFC tissues were removed from the −80°C freezer and placed on ice. Tissue was homogenized with a dounce homogenizer. The nuclear extract was prepared from the homogenized samples as per manufacturer's instructions using a CHEMICON ®Nuclear Extraction Kit (Millipore; Billerica, MA, USA). Protein concentrations were determined in the nuclear extracts using the Pierce BCA Protein Assay kit (Pierce Biotechnology, Inc., Rockford, IL, USA). Nuclear fractions were then assayed for Fos protein levels using the Pierce c-Fos Transcription Factor Kit (Pierce Biotechnology, Inc., Rockford, IL, USA), according to the manufacturer's instructions. A Wallac 1420 VICTOR luminometer (PerkinElmer; Waltham, MA, USA) was used to measure chemiluminesence in the ELISA plates. Data were analyzed and plotted using GraphPad Prism (GraphPad Software, Inc.; La Jolla, CA, USA).

### Rat apparatus and training

Sixteen operant-conditioning chambers (30.5 × 24.1 × 29.2 cm; MED Associates, St. Albans, VT) were utilized in conducting the DRL experiments. Each chamber was enclosed in a melamine sound-attenuating cubicle. A white noise generator provided masking noise. The interior of each chamber consisted of three levers mounted on one wall with a house light mounted on the opposite wall. The house light was turned on at initiation of each test session and was turned off at the termination of each session. A water access port was situated next to the lever in the middle of the wall, wherein a reinforced response caused a clicker apparatus to sound paired with a dipper (0.02-ml cup) to be lifted from a water trough to an opening in the floor of the access port for 4 s.

Rats were water deprived for ~22.5 h before each session. Rats were initially trained under an alternative fixed ratio 1 water reinforcement schedule with a fixed 1-min time schedule for automatic reinforcement. Thus, each response was reinforced, with water also provided every minute in the absence of a response. Rats that did not acquire lever-pressing behavior following three daily 1-h sessions under this schedule were trained using the method of successive approximation. Following acquisition of lever-pressing behavior, rats were trained daily on DRL 18-s sessions for ~2 weeks, following which they were advanced to DRL 72-s sessions. Responding on these sessions became stable after ~8 weeks. Experimental test sessions lasted for 1 h and were conducted 5 days/week during light hours.

### Mouse apparatus and training

Twelve operant-conditioning chambers (30.5 × 24.1 × 29.2 cm; MED Associates, St. Albans, VT) were used for the DRL experiments. The levers in these chambers were mounted on one wall with an adjacent food magazine next to the lever in the middle of the wall. A reinforced response caused a clicker apparatus to activate and the pellet feeder to deposit one 45-mg sucrose pellet (BioServ, Frenchtown, NJ) into the food magazine. The house light, which was mounted on the ceiling, was turned on when the session began, remained on throughout the entire session, and was turned off at the end of the session. Each experimental chamber was enclosed in a melamine sound-attenuating cubicle and equipped with a white noise generator to provide masking noise.

Each mouse was initially trained under a variable time 60-s operant schedule for 5 days, followed by fixed ratio-1 ratio schedule for 5 days. After the mice had acquired lever-pressing behavior, they were trained during daily DRL 6-s sessions for ~1 week, then mice progressed through weekly ascending DRL requirements (12, 18, 24, 30 s) before moving to DRL 36-s sessions. The responding on these sessions became stable after ~6 weeks. Experimental sessions lasted for 45 min and were conducted 5 days/week during light hours.

### Delayed reinforcement of low-rate (DRL) data analyses

Drugs were administered to the animals once or twice weekly with at least 1 week between subsequent drugs to minimize possible carryover effects. Drug treatments were administered on Tuesdays and Fridays. No treatments were administered on other test days. Wednesdays were control days. “Control” behavior was calculated as the pooled mean lever presses or reinforcers received during Wednesday sessions across all Wednesdays for the duration of each study. Performance was normalized to percent of control by dividing total responses (or reinforcers) for each drug or vehicle treatment by the mean number of responses (reinforcers) in the respective pooled control condition, multiplied by 100.

The data analyses were conducted on the raw response and reinforcement data. All behavioral data are expressed as the mean ± s.e.m. normalized to the control condition. The effects of LSN2424100 (3–40 mg/kg), SB334867 (3–40 mg/kg), almorexant (10–100 mg/kg) along with its inactive enantiomer (100 mg/kg), and imipramine (1–15 mg/kg) were analyzed using separate One-Way repeated measures ANOVAs on responses and reinforcements, followed by Dunnett's *post-hoc* tests (alpha = 0.05).

## Results

### Radioligand binding and intracellular calcium mobilization

Using both an orexin filtration binding assay and a functional intracellular calcium mobilization assay performed in antagonist mode, LSN2424100 demonstrated selectivity for human OX2 receptors stably expressed in HEK293 cells over OX1 receptors (Table 1). LSN2424100 was approximately 87-fold more potent at OX2 with a *Ki* of 4.49 nM (OX1 *Ki* = 393 nM) in the receptor binding assay. In the functional assay, LSN2424100 was over 200-fold more potent for human OX2 with a *Kb* = 0.44 nM whereas the *Kb* for human OX1 was 90.3 nM. Similar results were obtained in the rat OX receptor cell lines, with rat OX2 and OX1 *Kb* = 0.83 and 175 nM, respectively, indicating 210-fold selectivity. Conversely, SB334867 was more selective for the human OX1 receptor (Table 1). SB334867 was completely inactive at the OX2 receptor in both the *in vitro* assays. The measured binding affinity of SB334867 for the OX1 receptor was 173 nM. SB334867 showed potent inhibition of intracellular calcium mobilization, with OX1 *Kb* in human and rat = 8.68 and 7.1 nM, respectively. In addition to testing selective orexin agents, we also evaluated the non-selective orexin antagonist, almorexant. The more active S-enantiomer of almorexant demonstrated balanced activity for both human OX1 and OX2 receptors (Table 1). The binding affinities of (S)-almorexant for OX1 and OX2 receptors were 21 and 6.9 nM, respectively. Similarly, in the functional assay, (S)-almorexant showed roughly equal potency at inhibiting intracellular calcium mobilization in cells expressing hOX1 and hOX2 receptors, with *Kb*-values of 2.32 and 1.73 nM, respectively. The (R)-enantiomer of almorexant was inactive in both assays.

**Table 1:** ***In vitro* binding and functional activity at human recombinant OX1 and OX2 receptors**.

**Compound**	**Receptor binding affinity, *Ki*(nM)^a^**		**Functional antagonist activity, *Kb* (nM)^b^**			
	**OX1**	**OX2**	**hOX1**	**hOX2**	**rOX1**	**rOX2**
LSN2424100	393 ± 47 (3)	4.49 ± 1.39 (3)	90.3 ± 17.7 (2)	0.44 ± 0.11 (3)	175 ± 41 (2)	0.83 ± 0.19 (2)
SB334867	173 ± 11 (3)	>10,000 (3)	8.68 ± 1.76 (3)	>10,000 (3)	7.1 ± 0.71 (3)	>10,000 (3)
Almorexant (S)	21 ± 3.2 (2)	6.9 ± 0.18 (2)	2.32 ± 0.18 (3)	1.73 ± 0.35 (3)	3.2 ± 0.7 (4)	4.4 ± 1.6 (4)
Almorexant (R)	>10,000 (2)	>10,000 (2)	>10,000 (2)	>10,000 (2)	NT	NT

a Radioligand binding was performed using HEK293 membranes expressing either human OX1 or OX2 receptors in the presence of [^125^I]-orexin A with a 90 min incubation time. Data represent the mean ± s.e.m. performed on separate occasions with the number of independent experiments in parenthesis. (R) is the inactive enantiomer of almorexant.

b Inhibition of calcium mobilization from HEK293 cells stably expressing human or rat OX1 or OX2 receptors, respectively. Functional antagonist activity was determined by measuring the effects of an EC_70_ concentration of orexin-A. Data represent the mean ± s.e.m. performed on separate occasions with the number of independent experiments in parenthesis. NT = not tested.

### Rat exposure and unbound fraction

Pharmacokinetic data for LSN2424100 in rats are summarized in Table 2. LSN2424100 exhibits good exposure in Sprague–Dawley rats after IP, but not PO, administration of a 30 mg/kg dose (AUC_0–6 *h*_ = 1300 and 269 ng^*^h/mL, respectively). Mean maximum plasma concentrations observed following a single 30 mg/kg IP and PO dose were 1170 and 196 ng/ml, respectively at 0.5 h post injection. Mean brain concentrations observed following a single 30 mg/kg IP and PO dose were 77.2 and 33.5 ng/g, respectively, at 2 h post injection. Unbound fraction in plasma and brain were determined to be 0.0341 and 0.0089, respectively.

**Table 2:** **Pharmacokinetic datafor LY2424100^*a*^**.

**Parameter^b^**	**30 mg/kg IP**	**30 mg/kg PO**
AUC_0–6 *h*_ (ng*h/ml)	1300	269
*C*_max_ (ng/ml)	1170	196
*t*_max_ (hr)	0.5	0.5
Brain concentration^*c*^ (ng/g)	77.2	33.5
Plasma concentration^*c*^ (ng/ml)	149	45.8
Brain:plasma ratio^*c*^	0.52	0.72
*F_u_* (brain/plasma)	0.0089/0.0341	
*C^c^_u_* (brain/plasma, nM)	5.93/2.99	0.67/3.52

a n = 2 cannulated rats/dose.

b Abbreviations: AUC_0–6 h_, area under the concentration vs. time curve from time 0–6 h; C_max_, maximum observed drug concentration; t_max_, time to maximum observed drug concentration; F_u_, fraction unbound in brain/plasma; C_u_ (unbound or free concentration) = C_total_ × F_u_.

c Two hours post treatment.

### c-Fos expression

**Figure 2:**
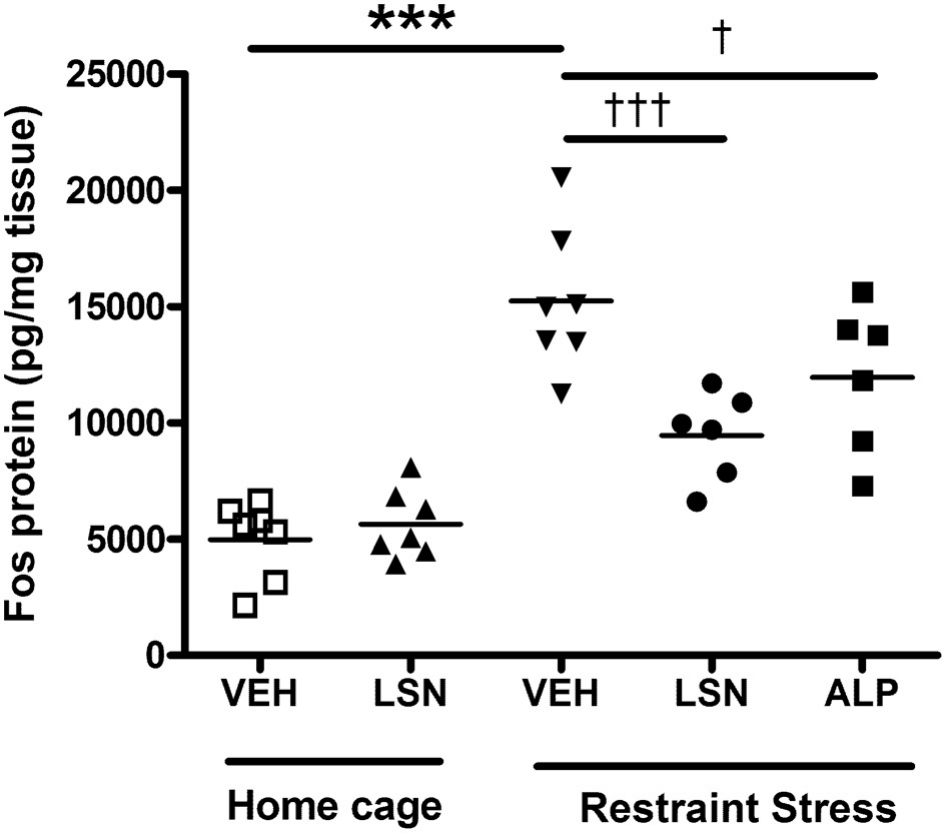
**c-Fos protein expression in rat prefrontal cortex with and without restraint stress**. In the absence of stress, LSN2424100 (*n* = 7) had no effect on c-fos levels compared to vehicle treated subjects (*p* > 0.05; *n* = 7). Restraint stress significantly increased c-fos levels in vehicle-treated rats (^***^p < 0.001; *n* = 7), and this effect was blocked by both LSN2424100 (^†††^*p* < 0.001; *n* = 6) and alprazolam (^†^*p* < 0.05; *n* = 6).

Administration of LSN2424100 to subjects in home cages produced no effects on c-fos protein levels in the rat prefrontal cortex, compared to vehicle treated subjects (*p* > 0.05; Figure 2). However, restraint stress invoked a significant increase in c-fos expression in vehicle treated rats [*F*_(4, 28)_ = 23.19; *p* < 0.001], and this effect was attenuated by pre-treatment with either LSN2424100 or alprazolam (*p* < 0.05; Figure 2).

### DRL

**Figure 3:**
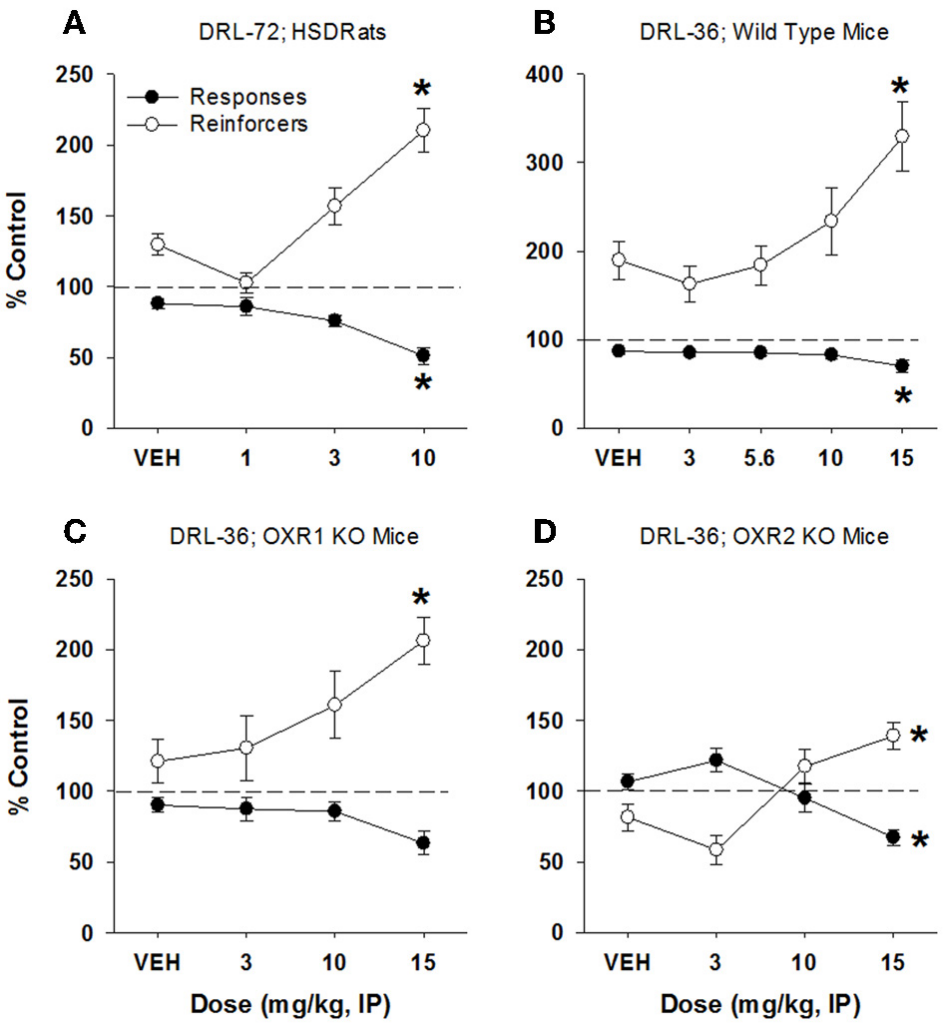
**Antidepressant-like effect of imipramine on DRL-72-s responding in rats and DRL-36-s responding in mice**. Consistent with literature reports, imipramine produced a significant antidepressant-like effect in both rat DRL-72-s (**A**; *n* = 12) and mouse DRL-36-s models (**B**; *n* = 16), as demonstrated by a significant increase in reinforcers (*p* < 0.001 and *p* < 0.01, respectively) and significant decrease in total responses emitted (*p* < 0.01 and *p* < 0.001, respectively). Importantly, the antidepressant-like efficacy of imipramine was not altered in mice lacking OX1 or OX2 receptors (**C,D**, respectively; *n* = 8). ^*^p < 0.05 vs. respective vehicle, Dunnett's post-hoc analyses.

In rats, imipramine produced a significant antidepressant-like response, characterized by a decrease in total lever presses emitted (51% of control responding) at 10 mg/kg [*F*_(3, 68)_ = 10.16, *p* < 0.0001] with a concomitant increase in reinforcers received [210% of control responding; *F*_(3, 68)_ = 14.47, *p* < 0.001; Figure 3A]. Doses of 1 and 3 mg/kg had no significant effect on behavior. Raw (non-normalized) data for all DRL experiments are provided in Supplementary Table S1. In mice, imipramine also produced an antidepressant-like signature, with an increase in reinforcers received (330% of control, Figure 3B) at 15 mg/kg [*F*_(4, 60)_ = 7.32, *p* < 0.001], and a concomitant decrease in lever presses emitted [70% of control; *F*_(4, 60)_ = 4.2, *p* < 0.005]. The antidepressant-like effect of imipramine was maintained in mice lacking OX1 receptors, as demonstrated by a dose-dependent increase in reinforcers received up to 15 mg/kg [206% of control; *F*_(3, 44)_ = 3.33, *p* = 0.028; Figure 3C], and a decrease in lever presses emitted (63% of control) but this difference did not quite reach statistical significance [*F*_(3, 44)_ = 2.64, *p* = 0.06; Dunnett's *post-hoc* test, *p* = 0.02; Figure 3C]. Lower doses of imipramine did not affect lever pressing in mice lacking OX1 receptors. Similarly, imipramine produced antidepressant-like effects in mice lacking OX2 receptors, with a significant effect in both lever presses emitted [*F*_(3, 44)_ = 6.63, *p* < 0.001] and reinforcers received [*F*_(3, 44)_ = 7.58, *p* < 0.001; 70 and 140% of control, respectively, at 15 mg/kg; Figure 3D].

**Figure 4:**
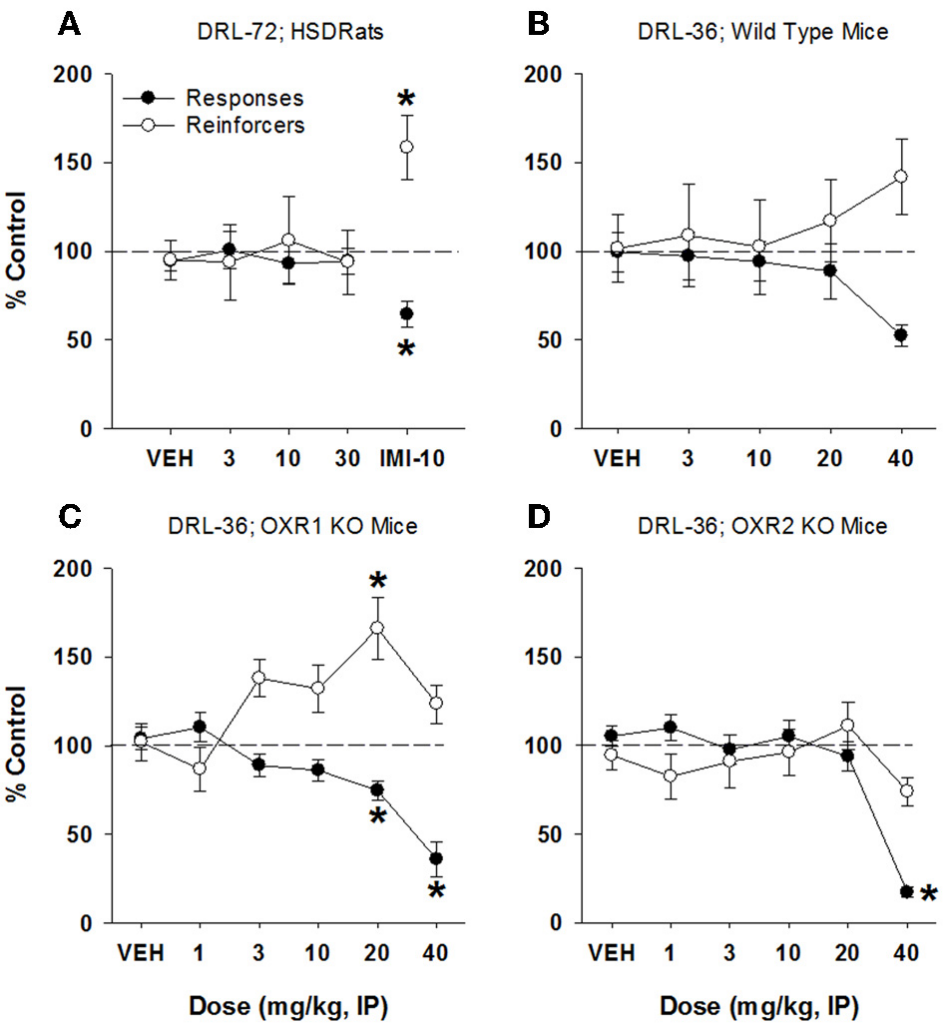
**SB334867 failed to produce antidepressant-like effects in rat DRL-72-s or mouse DRL-36-s. (A)** SB334867 did not affect total responses or reinforcers received in male SD rats maintained on a DRL-72-s schedule of responding within a dose range of 3–30 mg/kg (*n* = 7). Importantly, the positive control, imipramine, did produce an antidepressant-like effect in this experiment at 10 mg/kg; **(B)** no significant effect of SB334867 was observed on total responses emitted or reinforcers received in C57Bl/6 mice (*n* = 7); **(C)** significant decrease in total responses emitted by mice lacking OX1 receptors at 20 and 40 mg/kg (*p* < 0.01), with a concomitant increase in reinforcers received at 20 mg/kg (*p* < 0.01; *n* = 8); **(D)** significant decrease in total responses emitted (*p* < 0.05) without a concomitant increase in reinforcers received, by mice lacking OX2 receptors, at 40 mg/kg (*n* = 8). ^*^p < 0.05 vs. respective vehicle, Dunnett's post-hoc analyses.

**Figure 5:**
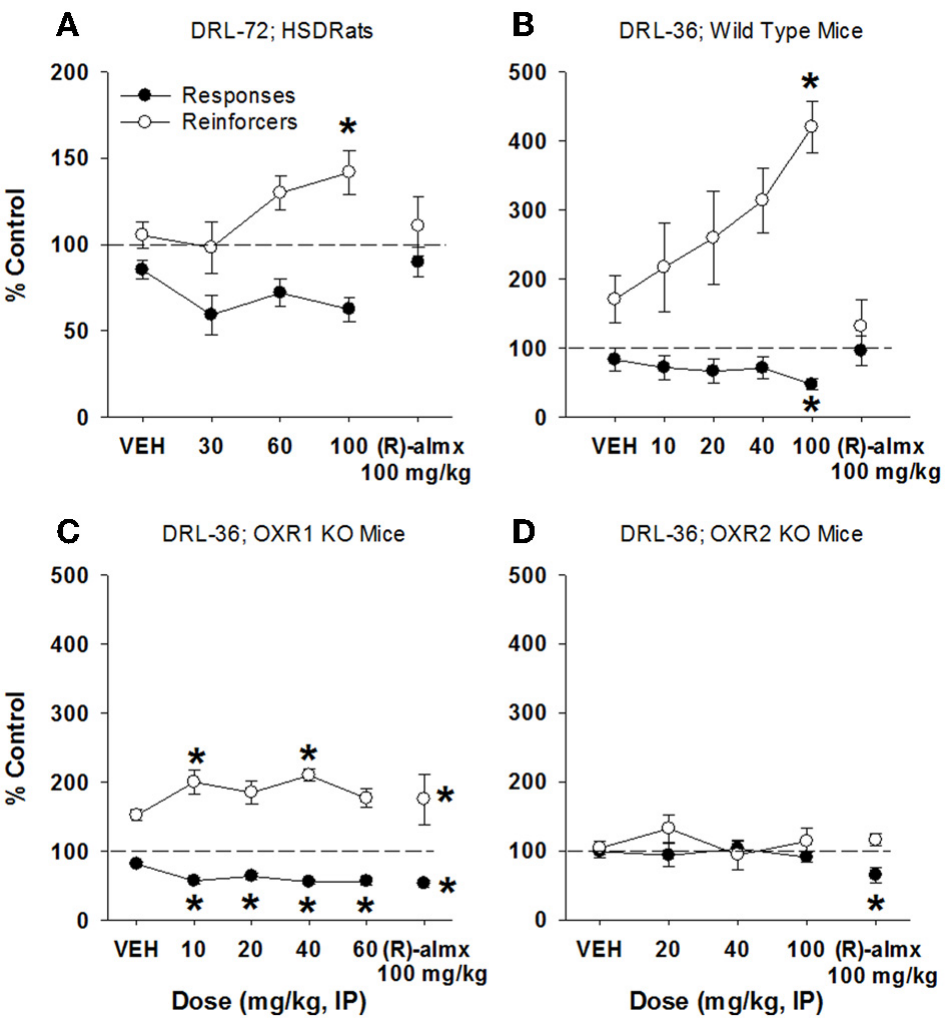
**Antidepressant-like effect of almorexant on DRL-72-s responding in rats and DRL-36-s responding in mice**. **(A)** dose-dependent increase in the number of reinforcers obtained by male SD rats in DRL-72-s, with significant effects at 100 mg/kg (*p* < 0.05), as well as a lack of effect of the inactive enantiomer at 100 mg/kg (*n* = 11); **(B)** dose-dependent and significant increase in the number of reinforcers obtained (*p* < 0.05) by C57Bl/6 mice in DRL-36-s at 100 mg/kg and a significant decrease (*p* < 0.05) in total responses emitted at 100 mg/kg, as well as lack of effect of the inactive enantiomer at 100 mg/kg (*n* = 8); **(C)** significant decrease in responses emitted at all doses tested (*p* < 0.05) with a concomitant increase in reinforcers received at doses of 10 and 40 mg/kg (*p* < 0.05) in mice lacking OX1 receptors (*n* = 8). However, 100 mg/kg of the inactive enantiomer also produced significantly fewer responses (*p* < 0.05) with a concomitant increase in reinforcers received (*p* < 0.05) in mice lacking OX1 receptors; **(D)** almorexant failed to produce antidepressant-like effects in mice lacking OX2 receptors, up to 100 mg/kg (*n* = 8). The inactive enantiomer (100 mg/kg) significantly reduced total responses emitted in mice lacking OX2 receptors (*p* < 0.05), without significantly affecting reinforcers earned (*n* = 8). ^*^p < 0.05 vs. respective vehicle, Dunnett's post-hoc analyses.

In rats, SB334867 did not produce a significant change in either reinforcers received or total lever presses made, at doses up to 30 mg/kg (Figure 4A). Importantly, the positive control used in this test, imipramine, significantly increased reinforcers received [160% of control, *F*_(4, 24)_ = 9.22, *p* < 0.001] and decreased lever presses [64% of control, *F*_(4, 24)_ = 16.21, *p* < 0.001]. In mice, SB334867 did not significantly affect lever presses emitted or reinforcers received up to 40 mg/kg (*p* > 0.05; Figure 4B). In mice lacking OX1 receptors, SB334867 produced a significant decrease in lever presses emitted at 20 and 40 mg/kg (75 and 35% of control, respectively; *F*_(5, 58)_ = 10.5, *p* < 0.001), while only producing a significant increase in reinforcers received at 20 mg/kg [166% of control; *F*_(5, 58)_ = 3.95, *p* < 0.005; Figure 4C]. Although an increase reinforcers received was observed at 40 mg/kg (123%), this was not significant. In mice lacking OX2 receptors, SB334867 only produced a disruption of lever pressing at 40 mg/kg [*F*_(5, 58)_ = 17.79, *p* < 0.001; Figure 4D]. At this dose, lever presses emitted were less than 20% of control, while reinforcers received were 75% of control, indicative of the animals' inability to behave within the scheduled operant parameters.

(S)-Almorexant treatment in rats produced a significant increase in reinforcers received [*F*_(3, 30)_ = 4.09, *p* < 0.02; Figure 5A], while the reduction in lever presses emitted did not reach statistical significance (*p* > 0.05). The inactive enantiomer (R)-almorexant produced no effect on behavior at 100 mg/kg (*p*s > 0.05). In mice, (S)-almorexant produced a significant increase in reinforcers received [>400% of control; *F*_(4, 28)_ = 5.95, *p* = 0.001], while also producing a significant dose-related decrease in lever presses emitted [47% of control; *F*_(4, 28)_ = 6.46, *p* < 0.001; Figure 5B]. Again, (R)-almorexant produced no significant effects on behavior at 100 mg/kg. In mice lacking OX1 receptors, all doses of (S)-almorexant reduced the number of lever presses emitted [10–60 mg/kg; *F*_(4, 51)_ = 10.16, *p* < 0.001] while a significant increase in reinforcers received was observed at 10 and 40 mg/kg [*F*_(4, 51)_ = 4.39, *p* < 0.004; Figure 5C], although these effects do not appear to be dose-dependent. In these animals, (R)-almorexant also produced effects similar to (S)-almorexant, with significant increases in reinforcers received and a concomitant significant decrease in lever pressing [*t*_(54)_ = 2.04, *p* < 0.05 and *t*_(54)_ = 2.04, *p* < 0.05, respectively]. In mice lacking OX2 receptors, the effect of (S)-almorexant was completely abolished (*p*s > 0.05; Figure 5D). In these mice, (R)-almorexant (100 mg/kg) produced a significant decrease in lever presses emitted [65% of control; *t*_(54)_ = 2.04, *p* < 0.05], but did not significantly affect the number of reinforcers received.

**Figure 6:**
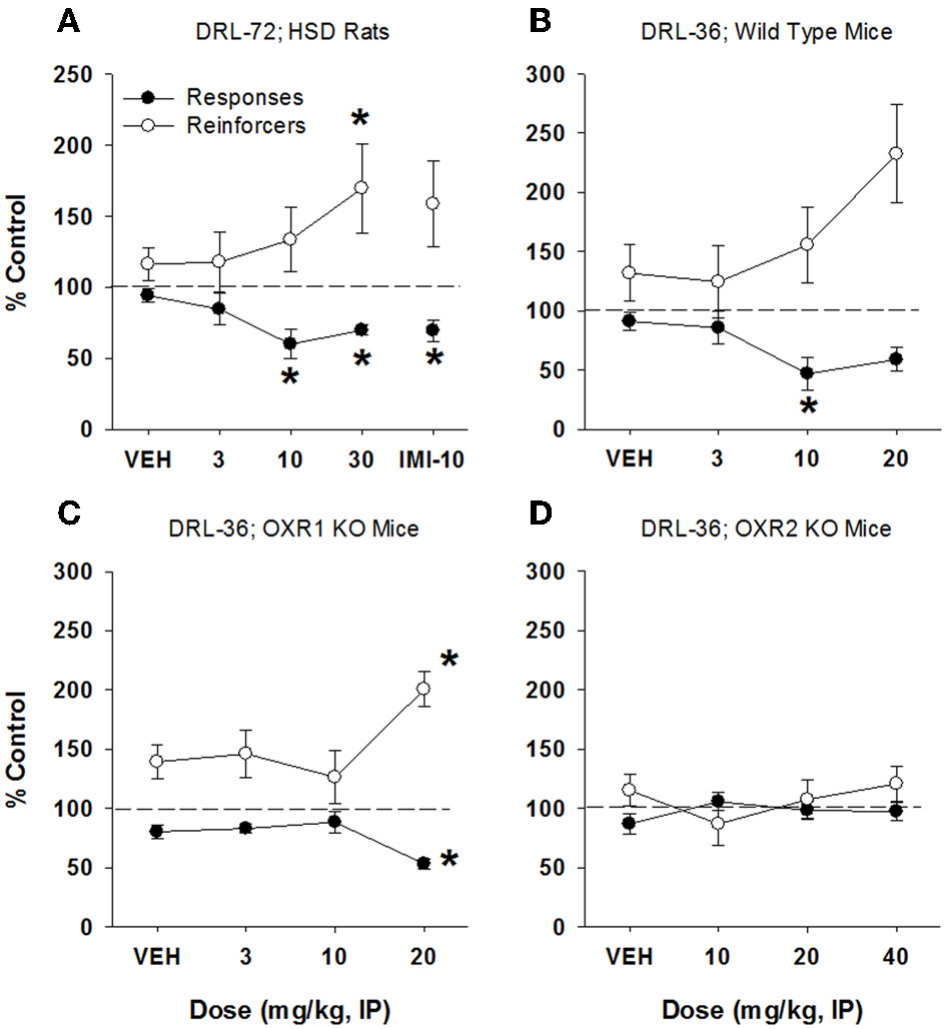
**Antidepressant-like effects of LSN2424100 on DRL-72-s responding in rats and DRL-36-s responding in mice**. **(A)** dose-dependent increase in the number of reinforcers obtained by male SD rats in DRL-72-s (*n* = 7), with a significant effect at 30 mg/kg (*p* < 0.05) and a significant decrease in total responses emitted at 10 and 30 mg/kg (*p* < 0.05); **(B)** a significant decrease in total responses emitted by C57Bl/6 mice in DRL-36-s at 10 mg/kg (*p* < 0.05; *n* = 8); **(C)** antidepressant-like efficacy of LSN2424100 is maintained in mice lacking OX1 receptors, with a significant increase in reinforcers obtained and a significant decrease in responses at 20 mg/kg (*p* < 0.05; *n* = 8); **(D)** antidepressant-like effect of LSN2424100 is lost in mice lacking OX2 receptors, as illustrated by lack of significant effects in either responses emitted or reinforcers received at all doses (*n* = 8). ^*^p < 0.05 vs. respective vehicle, Dunnett's post-hoc analyses.

When administered to rats, LSN2424100 produced a significant increase in reinforcers received [Figure 6A; 170% of control; *F*_(4, 24)_ = 3.13, *p* = 0.033] at 30 mg/kg, along with a significant decrease in lever presses made [60 and 70 of control; *F*_(4, 24) = 4.64_, *p* = 0.006] at 10 and 30 mg/kg, respectively. In mice, LSN2424100 produced an increase in reinforcers received (up to 225% of control, Figure 6B), but this effect did not reach statistical significance. LSN2424100 treatment did produce a significant decrease in lever presses emitted at 10 mg/kg [50% of control; *F*_(3, 35)_ = 3.28, *p* = 0.032]. A higher dose of 20 mg/kg produced a decrease in lever presses emitted, but this result did not reach statistical significance (Figure 6B). In mice lacking OX1 receptors, the antidepressant-like efficacy of LSN2424100 was maintained, as demonstrated by a statistically significant increase in reinforcers received at 20 mg/kg [200% of control; *F*_(3, 36) = 2.98_, *p* = 0.044], and a significant decrease in lever presses emitted at this dose [50% of control; Figure 6C; *F*_(3, 36)_ = 4.73, *p* = 0.007]. In mice lacking OX2 receptors, however, there was no significant change either in reinforcers received or lever presses emitted with LSN2424100 doses administered up to 40 mg/kg (Figure 6D), indicating complete loss of antidepressant-like efficacy in OX2 knockout mice.

## Discussion

Herein, we describe the pharmacological characterization of a structurally-novel OX2 receptor antagonist, LSN2424100, with high affinity and selectivity for OX2 over OX1 receptors. *In vitro* selectivity data for OX2 over OX1 receptors obtained using radioligand binding and intracellular calcium mobilization assays showed 87- and 205-fold selectivity, respectively (210-fold selectivity in the rat functional assay). Results showed that the two comparator compounds, SB334867 and (S)-almorexant, possessed *in vitro* profiles in the expected ranges, wherein SB334867 showed high affinity and selectivity for OX1 receptors, while the dual orexin antagonist, almorexant, showed roughly equal affinity for OX1 and OX2 receptors. Pharmacokinetic data indicated that LSN2424100 exhibits good exposure in male Sprague–Dawley rats after IP, but not PO, administration of a 30 mg/kg dose (AUC_0–6 *h*_ = 1300 and 269 ng^*^h/mL, respectively), suggesting its use as a tool compound to evaluate the role of OX2 receptor signaling mechanisms in preclinical models.

Here, we report that orexin-2 receptor antagonists, including LSN2424100 and almorexant, exhibit antidepressant-like efficacy in the rat DRL-72 sec model, at 20–30 and 100 mg/kg, respectively, as indicated by significant reductions in total lever presses emitted along with concomitant increases in reinforcers received. Our data are consistent with previous reports that increased OX signaling produced anhedonia-like symptoms in rats (measured by intracranial self-stimulation) (Boutrel et al., 2005), whereas almorexant produced antidepressant-like effects in a model of chronic unpredictable mild stress (Nollet et al., 2012). Interestingly, Nollet et al. (2011) demonstrated that chronic unpredictable mild stress increased activation of dorsomedial hypothalamus/perifornical area (DMH-PFA), where orexin cell bodies are localized. Moreover, they reported that chronic treatment with the SSRI, fluoxetine, reversed the DMH-PFA activation, suggesting a role for orexin signaling in the pathophysiology of depression and antidepressant treatment response (Nollet et al., 2011). The OX1 antagonist SB334867 did not produce antidepressant-like effects in the assays studied here. Together, the pharmacological data suggest that OX2 receptor signaling may be involved in modulating mood states. The results presented here contradict other studies suggesting that increases in orexin-dependent signaling may produce antidepressant-like effects (Ito et al., 2008; Lutter et al., 2008; Scott et al., 2011). The reason for this difference is unclear, but may be related to methodological differences between labs. Efficacy in the forced swim test, although useful in many respects, does not always translate to clinical antidepressant efficacy (De Pablo et al., 1989). The DRL model used here has strong construct and predictive validity, and is less likely to produce false positive results (O'Donnell et al., 2005; Zhang et al., 2009). Although less susceptible to false positive results due to motor effects, drugs that dramatically affect food intake, as has been reported for orexin antagonists (Mieda et al., 2006; Boutrel et al., 2010), can affect behavior in the DRL model. However, LSN2424100 was found to have no direct effects on food or water intake (Fitch et al., unpublished results; and see Anderson et al., under review). Alternatively, differences between our study and previous reports may be related to the use of mice lacking the orexin peptide (Lutter et al., 2008) vs. mice lacking individual OX1 or OX2 receptors (current study).

In addition to demonstrating antidepressant-like efficacy of OX2 antagonists in the rat DRL-72 s model, we extend the findings to demonstrate efficacy in a mouse DRL-36 s schedule of reinforcement. Importantly, we demonstrate similar antidepressant-like efficacy of imipramine in the mouse that closely resembles effects observed in the rat DRL model. In the mouse DRL model, LSN2424100 and almorexant produced antidepressant-like effects, similar to the effects observed in rats (Figures 5, 6). Moreover, the antidepressant-like effects of LSN2424100 and almorexant were completely abolished in mice lacking OX2 receptors, confirming the critical role of OX2 receptor signaling in mediating mood and antidepressant-like efficacy of the orexin antagonists tested here. Importantly, the effects of imipramine were found to be similar in mice lacking either OX1 or OX2 receptors, although efficacy appeared to be blunted in mice lacking OX2 receptors (Figure 3D). It is possible that the antidepressant-like phenotype observed in mice lacking OX2 receptors (see non-normalized data in Supplementary Table S1) may be responsible for the apparent blunted efficacy in the normalized data. Nonetheless, the efficacy of imipramine was not lost in mice lacking OX2 receptors, indicating that the antidepressant-like effects of imipramine are not mediated through orexin signaling mechanisms. By extension, our data further suggest that the antidepressant-like efficacy of OX2 and dual orexin receptor antagonists are mechanistically distinct from monoamine-based antidepressants. Considering the sleep disturbances often experienced by depressed individuals, the known side effect of SSRIs to exacerbate or even produce sleep disruption in depressed patients, and the robust sleep-enhancing properties of orexin antagonists (Mayers and Baldwin, 2005; Riemann, 2007; Thase et al., 2010), it seems plausible that selectively inhibiting OX2 receptor signaling may provide additional clinical benefits by simultaneously improving mood and sleep.

The OX1 antagonist, SB334867, appeared void of antidepressant-like activity in rats and mice, although antidepressant-like effects were observed in mice lacking OX1 receptors. Our results differ from previous reports that SB334867 produced antidepressant-like efficacy in the mouse forced swim test and tail suspension test (Scott et al., 2011). It has also been reported that mice lacking OX1 receptors had an antidepressant-like phenotype, an effect which we failed to replicate (Fitch et al., unpublished observations). The reason for the apparent antidepressant-like efficacy of SB334867 in mice lacking OX1 receptors is not known, but could involve either compensatory upregulation of OX2 receptors in mice with constitutive deletion of OX1 receptors, or perhaps some off-target, non-orexin pharmacology of SB334867 that may be either amplified or unmasked in the absence of OX1 receptors. Indeed, off-target pharmacology of SB334867 has already been reported by others, including activity at targets that may produce antidepressant-like effects, such as monoamine transporters, norepinephrine transporter, and 5-HT2C receptors (Gotter et al., 2012). Thus, data from our lab supports previous suggestions that SB334867 should not be considered OX1-selective. At the highest dose of 40 mg/kg, SB334867 produced behavioral disruption in mice lacking OX2 receptors, as indicated by strong reductions in total responding and concomitant reductions in reinforcers received. The mechanism by which SB334867 disrupted behavior in these mice is not known, but may be related to compensatory upregulation of OX1 receptors in these mice, an increase in functional sensitivity of OX1 receptors in the absence of OX2 receptors, or some unidentified off-target pharmacology of SB334867. Further studies will be required to understand these effects in the knockout mice.

Stress is a major trigger of depression and relapse to recurrent depressive episodes (Mazure, 1998). In rats, restraint stress stimulates ventral tegmental area (VTA) dopamine cell activity and c-fos expression in the mPFC, an area with dense reciprocal connections to the VTA that is known to be disrupted in depression (Drevets et al., 2008; Valenti et al., 2011). We demonstrate here that restraint stress-induced c-fos activation in the PFC was significantly attenuated by pre-treatment with LSN2424100. Our data are consistent with previous studies demonstrating that restraint-stress induced mPFC activation, as measured by c-fos expression in both rats and mice (Radley et al., 2006; O'Mahony et al., 2010; Valenti et al., 2011), and extend those results to demonstrate that orexin signaling may be involved in mediating stress responses within corticolimbic circuits. Although we and others have reported c-fos activation in the PFC in response to restraint stress, others have reported that restraint stress did not produce robust c-fos activation of hypothalamic orexin cells (Furlong et al., 2009), suggesting that the orexin system may modulate stress responses in the PFC through indirect pathways rather than by direct connections from hypothalamic cells where orexin is synthesized. While the importance of the PFC in regulating mood is well-established, the potential role of orexin in modulating these circuits is unclear. Further studies will be required to explore the exact mechanism by which orexin signaling may modulate prefrontal cortical activation following restraint stress, which may involve modulation of dopaminergic and noradrenergic neurotransmission (Borgland et al., 2008; Del Cid-Pellitero and Garzon, 2011).

Elucidating the role of the orexin system in modulating the complex physiological and behavioral traits associated with psychiatric disorders, including depression, has been a complex undertaking, confounded by a dearth of selective pharmacological tools. With its suggested involvement in the regulation of vigilance states, feeding and energy homeostasis, drug-seeking behavior, and emotional processing (Mieda et al., 2006; Richards et al., 2008; Aston-Jones et al., 2009; Boutrel et al., 2010; Sinton, 2011; Nollet and Leman, 2013), the orexin system appears to play a critical role in maintaining equilibrium in the mammalian nervous system (Sinton, 2011). In pathological states related to mood, arousal, or consummatory behaviors, the orexin system may present opportunities for pharmacological intervention with potentially beneficial outcomes. Recent research has begun to shed light on the diverse signaling properties of orexins. For example, it is known that OX1 and OX2 receptors can couple, either directly or indirectly, to several G-protein effectors, including G_q_, G_i/o_, and G_s_, with the potential to confer diverse signaling properties of either OXA or OXB through various intracellular pathways including phospholipase C, adenylyl cyclase, phospholipase A2, and phospholipase D (reviewed recently by Leonard and Kukkonen, 2014). However, little is known about which molecular pathways mediate the diverse physiological effects of orexins reported in the literature. Research focused on elucidating these mechanisms will benefit from an array of tools providing precise control of component function, including OX2-selective antagonists, which have not been widely tested to date with regard to potential antidepressant-like efficacy. Pharmacological tools, in particular, afford the investigator with an accessible, fast-acting and straightforward methodology for manipulation of receptor functioning to gain insight into the dynamics of receptor-ligand interactions, downstream physiological processes, signaling mechanisms, and behavioral output in preclinical models.

We demonstrate here that LSN2424100 is a novel orexin receptor antagonist with high affinity and selectivity for OX2 receptors and favorable pharmacokinetics following IP doses up to 30 mg/kg, and propose its use as a structurally-unique, synthetically-accessible tool for probing the role of OX2-dependent signaling in rodent models. Moreover, we demonstrate that LSN2424100 blocks stress-induced c-fos activation in the mPFC and produces antidepressant-like efficacy in an established animal model of depression in both rats and mice. Moreover, the antidepressant-like efficacy of LSN2424100 was completely abolished in mice lacking OX2, but not OX1, receptors, indicating a critical role of OX2-receptor-mediated signaling. In conclusion, LSN2424100 represents a functional tool compound for the investigation of OX2-receptor-mediated function, particularly in mood disorders.

### Conflict of interest statement

All authors are employees of, and stockholders in, Eli Lilly and Company. Financial support for the research conducted in this manuscript was provided by Eli Lilly and Company.
