# Supplementary material for: LSN2424100: a novel, potent orexin-2 receptor antagonist with selectivity over orexin-1 receptors and activity in an animal model predictive of antidepressant-like efficacy
Source: Front Neurosci. 2014 Jan 28;8:5. doi: 10.3389/fnins.2014.00005 (PMC3904085; doi:10.3389/fnins.2014.00005)
Supplement: Supplementary file 1 [file DataSheet1.DOCX]

**Supplementary Table S1**

| **HSD rats** |  |  |  |  |  |  |  |
| --- | --- | --- | --- | --- | --- | --- | --- |
|  | **imipramine** | |  | **Lever Presses** | | **Reinforcers** | |
|  |  |  |  | **Mean** | **SEM** | **Mean** | **SEM** |
|  |  | control |  | 90.25 | 3.32 | 9.67 | 0.65 |
|  |  | vehicle |  | 79.56 | 3.32 | 12.53 | 0.71 |
|  |  | 1 |  | 77.50 | 5.75 | 9.92 | 0.67 |
|  |  | 3 |  | 68.42 | 3.58 | 15.17 | 1.27 |
|  |  | 10 |  | 46.17 | 5.24 | 20.33 | 1.51 |
|  |  |  |  |  |  |  |  |
|  | **SB334867** | |  | **Lever Presses** | | **Reinforcers** | |
|  |  |  |  | **Mean** | **SEM** | **Mean** | **SEM** |
|  |  | control |  | 76.05 | 6.89 | 11.71 | 1.54 |
|  |  | vehicle |  | 71.76 | 3.87 | 11.14 | 1.28 |
|  |  | 3 |  | 76.57 | 7.99 | 11.00 | 2.49 |
|  |  | 10 |  | 70.71 | 8.55 | 12.43 | 2.93 |
|  |  | 30 |  | 71.86 | 5.52 | 11.00 | 2.11 |
|  |  | IMI-10 |  | 49.00 | 5.66 | 18.57 | 2.09 |
|  |  |  |  |  |  |  |  |
|  | **almorexant** | |  | **Lever Presses** | | **Reinforcers** | |
|  |  |  |  | **Mean** | **SEM** | **Mean** | **SEM** |
|  |  | control |  | 72.12 | 3.77 | 13.79 | 1.17 |
|  |  | vehicle |  | 61.56 | 3.79 | 14.53 | 1.07 |
|  |  | 30 |  | 42.73 | 8.29 | 13.55 | 2.05 |
|  |  | 60 |  | 51.91 | 5.77 | 17.91 | 1.39 |
|  |  | 100 |  | 45.09 | 5.11 | 19.55 | 1.76 |
|  |  | 100inactive |  | 64.73 | 6.29 | 15.27 | 2.37 |
|  |  |  |  |  |  |  |  |
|  | **LSN2424100** | |  | **Lever Presses** | | **Reinforcers** | |
|  |  |  |  | **Mean** | **SEM** | **Mean** | **SEM** |
|  |  | control |  | 80.19 | 4.19 | 9.10 | 1.40 |
|  |  | vehicle |  | 75.62 | 3.72 | 10.57 | 1.08 |
|  |  | 3 |  | 67.86 | 8.92 | 10.71 | 1.95 |
|  |  | 10 |  | 48.14 | 8.14 | 12.14 | 2.04 |
|  |  | 30 |  | 56.00 | 2.88 | 15.43 | 2.88 |
|  |  | IMI-10 |  | 55.71 | 6.06 | 14.43 | 2.76 |

| **WT Mice** |  |  |  |  |  |  |  |
| --- | --- | --- | --- | --- | --- | --- | --- |
|  | **imipramine** | |  | **Lever Presses** | | **Reinforcers** | |
|  |  |  |  | **Mean** | **SEM** | **Mean** | **SEM** |
|  |  | control |  | 277.00 | 8.93 | 6.15 | 0.70 |
|  |  | vehicle |  | 241.23 | 10.44 | 11.67 | 1.31 |
|  |  | 3 |  | 235.56 | 10.73 | 10.00 | 1.27 |
|  |  | 5.6 |  | 235.75 | 9.97 | 11.31 | 1.35 |
|  |  | 10 |  | 228.94 | 13.35 | 14.38 | 2.32 |
|  |  | 15 |  | 194.13 | 19.05 | 20.25 | 2.39 |
|  |  |  |  |  |  |  |  |
|  | **SB334867** | |  | **Lever Presses** | | **Reinforcers** | |
|  |  |  |  | **Mean** | **SEM** | **Mean** | **SEM** |
|  |  | control |  | 167.55 | 12.39 | 15.75 | 2.58 |
|  |  | vehicle |  | 166.71 | 18.47 | 16.00 | 2.96 |
|  |  | 3 |  | 162.86 | 21.95 | 17.14 | 4.56 |
|  |  | 10 |  | 157.57 | 18.66 | 16.14 | 4.19 |
|  |  | 20 |  | 148.57 | 26.17 | 18.43 | 3.66 |
|  |  | 40 |  | 87.67 | 9.91 | 22.33 | 3.35 |
|  |  |  |  |  |  |  |  |
|  | **almorexant** | |  | **Lever Presses** | | **Reinforcers** | |
|  |  |  |  | **Mean** | **SEM** | **Mean** | **SEM** |
|  |  | control |  | 243.13 | 22.72 | 6.63 | 1.14 |
|  |  | vehicle |  | 201.88 | 16.33 | 11.38 | 2.36 |
|  |  | 10 |  | 174.25 | 17.77 | 14.00 | 3.85 |
|  |  | 20 |  | 161.13 | 17.39 | 15.50 | 4.45 |
|  |  | 40 |  | 172.38 | 15.95 | 20.25 | 2.86 |
|  |  | 100 |  | 114.88 | 7.78 | 27.13 | 2.23 |
|  |  | 100inactive |  | 233.00 | 20.86 | 11.00 | 3.35 |
|  |  |  |  |  |  |  |  |
|  | **LSN2424100** | |  | **Lever Presses** | | **Reinforcers** | |
|  |  |  |  | **Mean** | **SEM** | **Mean** | **SEM** |
|  |  | control |  | 178.75 | 11.32 | 12.04 | 1.66 |
|  |  | vehicle |  | 163.00 | 13.25 | 15.88 | 2.87 |
|  |  | 3 |  | 153.38 | 24.73 | 15.00 | 3.70 |
|  |  | 10 |  | 83.88 | 24.47 | 18.75 | 3.85 |
|  |  | 20 |  | 105.43 | 18.07 | 28.00 | 4.99 |

| **OX1 KO** |  |  |  |  |  |  |  |
| --- | --- | --- | --- | --- | --- | --- | --- |
|  | **imipramine** | |  | **Lever Presses** | | **Reinforcers** | |
|  |  |  |  | **Mean** | **SEM** | **Mean** | **SEM** |
|  |  | control |  | 203.21 | 11.61 | 15.08 | 1.78 |
|  |  | vehicle |  | 183.17 | 10.40 | 18.25 | 2.33 |
|  |  | 3 |  | 177.63 | 17.34 | 19.68 | 3.47 |
|  |  | 10 |  | 174.13 | 13.16 | 24.25 | 3.57 |
|  |  | 15 |  | 128.50 | 16.91 | 31.13 | 2.49 |
|  |  |  |  |  |  |  |  |
|  | **SB334867** | |  | **Lever Presses** | | **Reinforcers** | |
|  |  |  |  | **Mean** | **SEM** | **Mean** | **SEM** |
|  |  | control |  | 190.13 | 7.01 | 17.31 | 1.12 |
|  |  | vehicle |  | 197.63 | 12.02 | 17.67 | 1.85 |
|  |  | 1 |  | 209.75 | 15.46 | 15.00 | 2.10 |
|  |  | 3 |  | 169.00 | 12.18 | 23.88 | 1.81 |
|  |  | 10 |  | 163.63 | 11.21 | 22.88 | 2.27 |
|  |  | 20 |  | 141.88 | 10.39 | 28.75 | 2.97 |
|  |  | 40 |  | 68.13 | 18.16 | 21.38 | 1.87 |
|  |  |  |  |  |  |  |  |
|  | **almorexant** | |  | **Lever Presses** | | **Reinforcers** | |
|  |  |  |  | **Mean** | **SEM** | **Mean** | **SEM** |
|  |  | control |  | 195.71 | 9.18 | 16.50 | 1.22 |
|  |  | vehicle |  | 159.00 | 5.92 | 25.06 | 1.22 |
|  |  | 10 |  | 111.38 | 10.19 | 33.00 | 2.78 |
|  |  | 20 |  | 124.75 | 8.78 | 30.50 | 2.70 |
|  |  | 40 |  | 108.25 | 6.69 | 34.63 | 1.49 |
|  |  | 60 |  | 110.13 | 9.59 | 29.13 | 2.28 |
|  |  | 100inactive |  | 103.75 | 9.27 | 28.88 | 2.13 |
|  |  |  |  |  |  |  |  |
|  | **LSN2424100** | |  | **Lever Presses** | | **Reinforcers** | |
|  |  |  |  | **Mean** | **SEM** | **Mean** | **SEM** |
|  |  | control |  | 197.92 | 9.87 | 15.83 | 1.52 |
|  |  | vehicle |  | 158.88 | 11.90 | 22.06 | 2.21 |
|  |  | 3 |  | 164.38 | 8.00 | 23.13 | 3.13 |
|  |  | 10 |  | 174.75 | 18.23 | 20.00 | 3.52 |
|  |  | 20 |  | 105.25 | 8.07 | 31.75 | 2.33 |

| **OX2 KO** |  |  |  |  |  |  |  |
| --- | --- | --- | --- | --- | --- | --- | --- |
|  | **imipramine** | |  | **Lever Presses** | | **Reinforcers** | |
|  |  |  |  | **Mean** | **SEM** | **Mean** | **SEM** |
|  |  | control |  | 173.92 | 8.46 | 23.75 | 2.11 |
|  |  | vehicle |  | 185.29 | 10.16 | 19.33 | 2.22 |
|  |  | 3 |  | 211.63 | 14.58 | 13.88 | 2.40 |
|  |  | 10 |  | 165.25 | 17.20 | 27.88 | 2.78 |
|  |  | 15 |  | 116.88 | 9.81 | 33.00 | 2.26 |
|  |  |  |  |  |  |  |  |
|  | **SB334867** | |  | **Lever Presses** | | **Reinforcers** | |
|  |  |  |  | **Mean** | **SEM** | **Mean** | **SEM** |
|  |  | control |  | 159.50 | 7.75 | 23.63 | 1.97 |
|  |  | vehicle |  | 167.88 | 8.80 | 22.33 | 2.01 |
|  |  | 1 |  | 175.50 | 11.78 | 19.50 | 3.02 |
|  |  | 3 |  | 155.75 | 13.04 | 21.50 | 3.58 |
|  |  | 10 |  | 167.75 | 14.40 | 22.75 | 3.05 |
|  |  | 20 |  | 149.50 | 13.04 | 26.25 | 3.16 |
|  |  | 40 |  | 27.38 | 4.52 | 17.50 | 1.91 |
|  |  |  |  |  |  |  |  |
|  | **almorexant** | |  | **Lever Presses** | | **Reinforcers** | |
|  |  |  |  | **Mean** | **SEM** | **Mean** | **SEM** |
|  |  | control |  | 173.50 | 10.94 | 21.71 | 2.18 |
|  |  | vehicle |  | 172.54 | 15.06 | 22.63 | 2.13 |
|  |  | 20 |  | 162.63 | 28.98 | 28.75 | 4.34 |
|  |  | 40 |  | 179.25 | 17.45 | 20.50 | 4.72 |
|  |  | 100 |  | 158.13 | 12.81 | 24.75 | 4.29 |
|  |  | 100inactive |  | 112.88 | 18.60 | 25.13 | 1.91 |
|  |  |  |  |  |  |  |  |
|  | **LSN2424100** | |  | **Lever Presses** | | **Reinforcers** | |
|  |  |  |  | **Mean** | **SEM** | **Mean** | **SEM** |
|  |  | control |  | 162.75 | 8.00 | 21.75 | 2.06 |
|  |  | vehicle |  | 141.31 | 13.68 | 25.06 | 2.89 |
|  |  | 10 |  | 171.75 | 12.46 | 18.88 | 3.84 |
|  |  | 20 |  | 160.00 | 12.08 | 23.38 | 3.52 |
|  |  | 40 |  | 158.25 | 12.63 | 26.25 | 3.28 |
